# Genome-wide analysis emancipates genomic diversity and signature of selection in Altay white-headed cattle of Xinjiang, China

**DOI:** 10.3389/fgene.2023.1144249

**Published:** 2023-03-30

**Authors:** Jialei Chen, Yushu Wang, Xingshan Qi, Haijian Cheng, Ningbo Chen, Zulfiqar Ahmed, Qiuming Chen, Chuzhao Lei, Xueyi Yang

**Affiliations:** ^1^ Life Science College, Luoyang Normal University, Luoyang, China; ^2^ College of Animal Science and Technology, Northwest A&F University, Yangling, China; ^3^ Biyang Xianan Cattle Technology and Development Company Ltd., Biyang, China; ^4^ Shandong Key Lab of Animal Disease Control and Breeding, Institute of Animal Science and Veterinary Medicine, Shandong Academy of Agricultural Sciences, Jinan, China; ^5^ Key Laboratory of Swine Genetics and Breeding of Ministry of Agriculture and Rural Affairs, and Key Lab of Agricultural Animal Genetics, Breeding and Reproduction of Ministry of Education, Huazhong Agricultural University, Wuhan, China; ^6^ Faculty of Veterinary and Animal Sciences, University of Poonch Rawalakot, Shabestar, Pakistan; ^7^ College of Animal Science, Xinjiang Agricultural University, Urumqi, Xinjiang, China

**Keywords:** Altay white-headed cattle, indigenous cattle breeds, whole-genome sequencing, genetic structure, selection signatures

## Abstract

Altay white-headed cattle have not received enough attention for several reasons. Due to irrational breeding and selection practices, the number of pure Altay white-headed cattle has decreased significantly and the breed is now on the eve of extinction. The genomic characterization will be a crucial step towards understanding the genetic basis of productivity and adaptability to survival under native Chinese agropastoral systems; nevertheless, no attempt has been made in Altay white-headed cattle. In the current study, we compared the genomes of 20 Altay white-headed cattle to the genomes of 144 individuals in representative breeds. Population genetic diversity revealed that the nucleotide diversity of Altay white-headed cattle was less than that of indicine breeds and comparable to that of Chinese taurus cattle. Using population structure analysis, we also found that Altay white-headed cattle carried the ancestry of the European and East Asian cattle lineage. In addition, we used three different methods (*F*_ST_, θπ ratio and XP-EHH) to investigate the adaptability and white-headed phenotype of Altay white-headed cattle and compared it with Bohai black cattle. We found *EPB41L5*, *SCG5* and *KIT* genes on the list of the top one percent genes, these genes might have an association with environmental adaptability and the white-headed phenotype for this breed. Our research reveals the distinctive genomic features of Altay white-headed cattle at the genome-wide level.

## 1 Introduction

The domestication of modern cattle was principally driven by two independent processes, one in the Fertile Crescent approximately 10,000 years ago, resulting in humpless taurine cattle (*Bos taurus*), whereas the other in the Indus Valley around 8,000 years ago, resulted in humped indicine cattle (*Bos indicus*) (Chen et al., 2018). These two subspecies are interfertile and have undergone historical and recent hybridization (Wu et al., 2018). Many cattle breeds adapted to harsh environmental conditions originate from interbreeding between the taurine and indicine populations (Verdugo et al., 2019). Historically, multiethnic livestock breeding backgrounds from Xinjiang national minorities spawned numerous native cattle breeds (Altay white-headed cattle and Kazakh cattle, etc.,), possibly including complicated genetic origins in Xinjiang (Liu et al., 2022). Geographically, Altay white-headed cattle were forced to adapt to the cold (annual average temperature 0.7°C–4.9°C) and dry (annual mean humidity 58.6%) climate due to the various landforms in the Altay prefecture of Xinjiang (Liu et al., 2022). In the current breeding context, poor socioeconomic rewards encourage this cattle breed to decline. Hence, a comprehensive exploration about the genetic diversity and adaptability of Altay white-headed cattle is needed.

Altay white-headed cattle are moulded by multicultural zones and complicated natural ecological environments (Svishcheva et al., 2020; Liu et al., 2022) and are preferred by the majority of herders in Xinjiang due to exceptional features such as cold temperature endurance, adaptability and disease resistance (Liu et al., 2022). It is native cattle breed that dates back a long time and is known for its outstanding production, attractiveness and gentle nature (Liu et al., 2022). The white-head phenotype is the most noticeable physical trait of Altay white-head cattle, distinguishing it from other local cattle breeds in China. Chinese indigenous cattle breeds are typically dark yellow to brown, with deeper markings on the head and neck, hooves and hindquarters, and lack the white spotting or piebald phenotypes observed in domesticated cattle (Ma et al., 2022). There are currently 3,900 Altay white-headed cattle, of which 3,100 are mainly in Buerjin County and 800 in Habahe County (Liu et al., 2022). An earlier study on Altay white-headed cattle using 100 K SNP markers demonstrated that Altay white-headed cattle had low heterozygosity, a high inbreeding degree, few families and significant differences in the number of individuals in each family (Liu et al., 2022). However, single nucleotide polymorphism (SNP) array data with only a few well-known SNPs may make it difficult to uncover important genetic information.

“Selection signatures” are the distinctive genetic traces or prints left in the areas of the genome that were exposed to selection (Nielsen, 2005; Jensen et al., 2016; Saravanan et al., 2020; Panigrahi et al., 2022). Identification of the selection features, such as disease, pests, drought, high temperature and high-altitude tolerance (Yang et al., 2016; Kim et al., 2017; Taye et al., 2017) can offer various livestock populations a selective advantage. Whole-genome sequencing (WGS) has dramatically improved our ability to detect the genomic regions under selection in livestock species (Panigrahi et al., 2022; Saravanan et al., 2022). We can now more easily identify the genomic areas being selected in livestock animals thanks to WGS. Previous studies identified several genes related to coat colour and extreme adaptation of livestock by using WGS (Kim et al., 2016; Wang et al., 2020; Shen et al., 2021). However, there has not been any research on how Altay white-head cattle adapt to extreme conditions.

Although research on the genetic diversity and lineage origins of local cattle breeds is becoming increasingly common, there are scant reports about the genomic variance in Altay white-headed cattle in the literature. To fill this gap, in the present study, we used whole-genome re-sequencing to determine the genetic diversity and signature of the selection of Altay white-headed cattle.

## 2 Materials and methods

### 2.1 Ethics statement

All experimental procedures with cattle used in the present study were approved by the Experimental Animal Manage Committee (EAMC) of Northwest A&F University (2011–31,101,684). All operations and experimental procedures complied with the National Standard of Laboratory Animals Guidelines for Ethical Review of Animal Welfare (GB/T 35892–2018) and Guide for the Care and Use of Laboratory Animals: Eighth Edition Consent for publication.

### 2.2 Sample collection and genome sequencing

We obtained ear tissue from 20 unrelated Altay white-headed cattle from the core breeding tracts of Altay, Xinjiang Province, China. Genomic DNA was extracted from the samples using a standard phenol–chloroform protocol (Toni et al., 2018). Paired-end libraries were constructed for each individual (350 bp insert size) and sequenced, using the Illumina-Nova 6000 Platform with a 2 × 150 bp model. Additionally, we used 144 publicly available genomes from NCBI, including Bohai cattle (n = 9), Brahman cattle (n = 4), Charolais cattle (n = 13), Hereford cattle (n = 13), Holstein cattle (n = 22), Kazakh cattle (n = 9), Mongolian cattle (n = 17), Simmental cattle (n = 16), Wannan cattle (n = 5) and Yanbian cattle (n = 16) (Supplementary Table S1).

### 2.3 SNP calling

Raw sequencing reads were processed to trim low-quality and adaptor sequences using the Trimmomatic tool (LEADING: 20 TRAILING: 20 SLIDING WINDOW: 3:15 AVGQUAL: 20 MINLEN: 35 TOPHRED33) (Bolger et al., 2014). Clean reads were aligned to the *Bos taurus* reference genome (ARS-UCD1.2) (Zimin et al., 2009) using the Burrows-Wheeler Aligner (BWA) program with the default parameters (Li and Durbin, 2009). Picard Tools (v.1.106) (http://broadinstitute.github.io/picard/) was used to generate quality matrices, and the Genome Analysis Toolkit (GATK, v.3.8) pipeline was employed for mapping (Nekrutenko and Taylor, 2012). We used the “HaplotypeCaller”, “GenotypeGVCFs” and “SelectVariants” arguments of GATK to call raw SNPs (McKenna et al., 2010) and generate VCF files. After SNP calling, we used “VariantFiltration” to obtain high-quality SNPs with the following parameters: QualByDepth (QD) < 2.0, FisherStrand (FS) > 60.0, RMS Mapping Quality (MQ) < 40.0, Mapping Quality Rank Sum Test (MQRankSum) < −12.5, Read Pos Rank Sum Test (ReadPosRankSum) < −8.0, and StrandOddsRatio (SOR) > 3.0. Finally, we used SnpEff (Yen et al., 2017) to annotate the functions of the SNPs based on the ARS-UCD1.2 database.

### 2.4 Genomic diversity

Runs of homozygosity (ROH) were detected across autosomes for each sample, using PLINK v.1.9 (Purcell et al., 2007) with the following settings: 1) required minimum density (-homozyg-density 50); 2) number of heterozygotes allowed in a window (-homozyg-window-het 3); and 3) number of missing calls allowed in a window (-homozyg-window-missing 5). The numbers and lengths of ROH in each breed were calculated, and the lengths of ROH were divided into three categories: 0.5–1 Mb, 1–2 Mb and 2–4 Mb (Zimin et al., 2009; Saravanan et al., 2021; Rajawat et al., 2022a; Rajawat et al., 2022b).

Linkage disequilibrium was calculated and visualized using Haploview v.4.1 (Barrett et al., 2005). The R package rehh v.3.01 (Gautier et al., 2017) was used to draw a haplotype bifurcation diagram that visualizes the breakdown of LD at increasing distances from the focal core allele (Sabeti et al., 2002). The haplotypes used to draw the bifurcation diagram were phased using Beagle v.4.1 (Browning and Browning, 2007).

### 2.5 Population genetic structure

The autosomal SNPs were pruned according to linkage disequilibrium (LD) using a 50 SNP window size, 5 SNP window shift and r^2^ of 0.1–1.0 in increments of 0.2. After quality control, we retained 7,786,318 SNPs. The filtered data were used to construct NJ tree, principal component analysis (PCA) and ancestry estimation. PCA was carried out using the smartPCA module of EIGENSOFT v.5.0 with default settings (Sabeti et al., 2002). Based on the pairwise genetic distance matrix using PLINK v.1.9 (Purcell et al., 2007), a neighbor-joining (NJ) tree was constructed by MEGA v.7.0 (Kumar et al., 2016), and the bootstrap value was set to 1,000. The genetic distance matrix was calculated by using the parameter “- distance matrix” of PLINK 1.9 and then the matrix was transformed into the “.meg” format, which was imported into MEGA v.7.0 software. Moreover, the phylogenetic tree was visualized using iTOL (Letunic and Bork, 2021). Finally, ancestry estimation was performed using ADMIXTURE with *K* = 2-4 to calculate the amount of admixture per individual (Alexander and Lange, 2011). The optimal *K* value was obtained according to the cross-validation (CV) value. Compared to other *K* values, *K* = 4 will exhibit low cross-validation errors. The *F*_ST_ was calculated using a sliding window approach (50 kb sliding window with 20 kb steps) and was applied to confirm the top signals. We also inferred a population-level phylogeny using the ML approach implemented in TreeMix (Pickrell and Pritchard, 2012). Local ancestry information was inferred using the software package RFMix in the Altay white-headed cattle (Maples et al., 2013). As population genetic structure and phylogenetic analysis revealed a genetic connection between Hereford cattle, Yanbian cattle and Altay white-headed cattle, Yanbian and Hereford were chosen as a reference panels. Based on the *Bos taurus* reference genome, BEDTools annotated the segments (Quinlan and Hall, 2010).

### 2.6 Genome-wide selection

For white-headed and non-white-headed cattle selection, we calculated the genome-wide distribution of the fixation index (*F*_ST_) using VCFtools (Danecek et al., 2011) with a sliding window of 50 kb and a 20 kb step size (da Silva Ribeiro et al., 2022). For cross-population extended haplotype homozygosity, we averaged the normalized XP-EHH score supplied by the norm module of selscan (Szpiech and Hernandez, 2014) with the same window and increment (Nielsen et al., 2007). For the XP-EHH selection scan, our test statistic was the average normalized XP-EHH score in each 50 kb region. Candidate genes under selection were defined as those overlapping sweep regions or within 20 kilobases (kb) of these signals. The top one percent of candidate windows identified by each method were considered potential candidate selection sweep regions.

### 2.7 Functional prediction analysis

Furthermore, candidate genes supported by three approaches were subjected to the Kyoto Encyclopedia of Genes and Genomes (KEGG) pathway and Gene Ontology (GO) analyses using KOBAS 3.0 (Bu et al., 2021) (http://kobas.cbi.pku.edu.cn/). We sed *Bos taurus* as an annotation background. Finally, the pathways (corrected *p*-value <0.05) were chosen as statistically significant terms.

## 3 Results

### 3.1 Genome resequencing and SNP identification

We mapped WGS data of 20 Altay white-headed cattle and the 144 published genomes to the *Bos taurus* reference genome (ARS-UCD1.2) (Supplementary Table S1). The average alignment rate and sequencing depth of the final set reached 98.87% and ∼16.09×, respectively (Supplementary Table S1). After quality control and SNP calling, we identified 48,841,721 SNPs for subsequent analysis. We annotated 20,427,430 SNPs ascertained in 20 Altay white-headed cattle by SnpEff software. After annotation, 11,592,427 SNPs were located in intergenic regions, and 45,511,117 were located in intronic regions. The results for other cattle breeds are shown in Supplementary Table S2. The Exons of Altay white-headed cattle contained 1.34% of the total SNPs, including 201,723 non-synonymous SNPs and 325,093 synonymous SNPs (Supplementary Table S2).

### 3.2 Population genetic diversity and relationships

**FIGURE 1:**
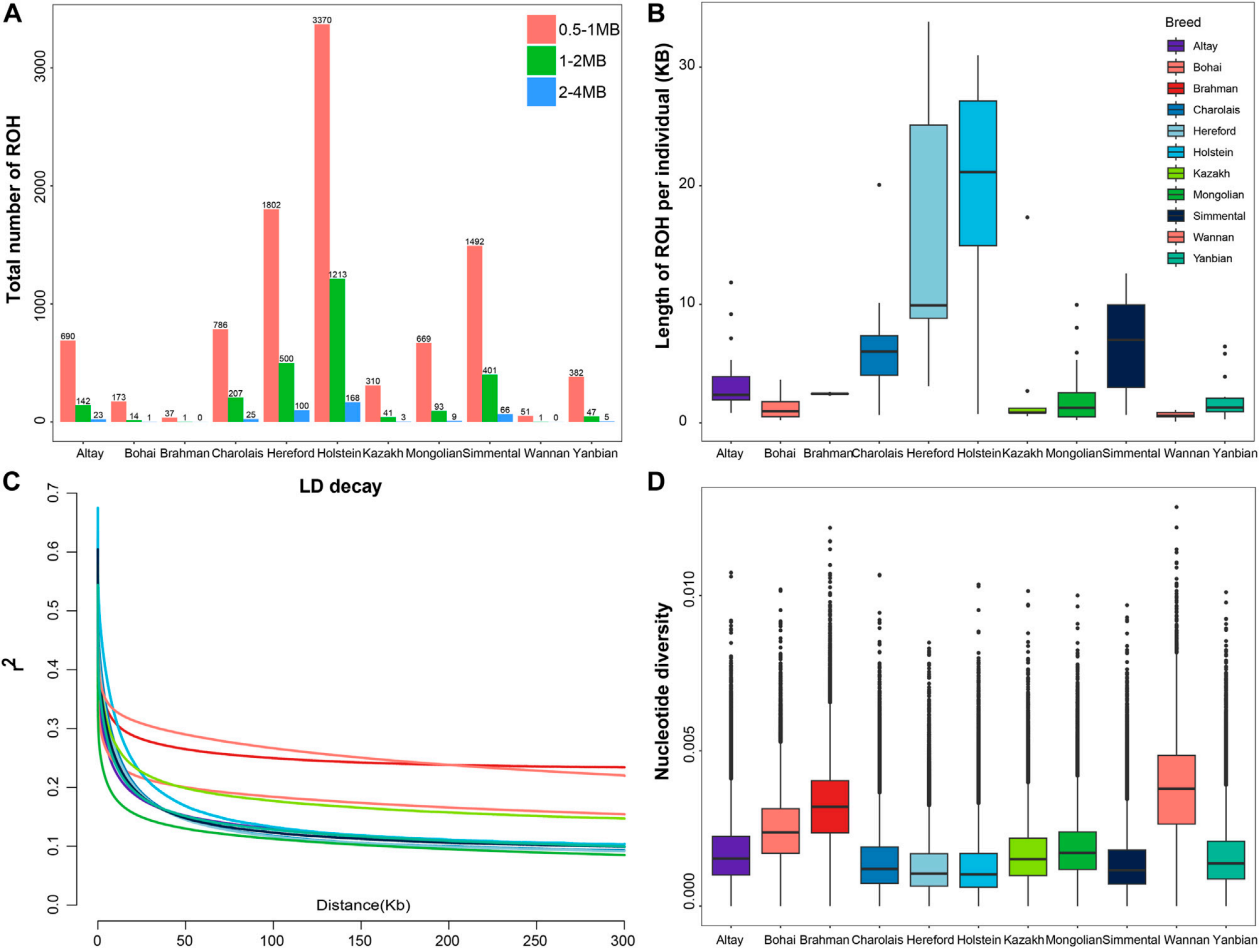
Summary of genomic variation statistics. ROH statistical distributions for 11 cattle breeds. **(A)** The number of ROH events in each breed. **(B)** The length of ROH events held by each breed. **(C)** Estimated genome-wide average LD decay from each breed. **(D)** Genome-wide nucleotide diversity distribution of each breed in 50 kb windows with 50 kb increments. The median of the distribution is shown by the horizontal line inside the box.; the box boundaries represent the first and third quartiles, and the points represent outliers. Outliers are data points that fall outside the whiskers.

We classified ROH into three categories according to length: 0.5–1 Mb, 1–2 Mb, and 2–4 Mb. Most of ROH observed in all cattle breeds were between 0.5 and 1 Mb in length. In European taurine cattle, medium (1–2 Mb) and long ROH (2–4 Mb) were found in Holstein, Hereford, Simmental and Charolais cattle (Figure 1A **;** Supplementary Table S3). Compared to other cattle populations, the total length of the ROH in Altay white-headed cattle was medium, somewhat shorter than in Simmental and Charolais cattle. In the shorter length category (0–5 Mb), no breeds of cattle with ROH lengths between 0 and 5 M were found, but in the longer length category (>40 Mb), the Simmental breed showed the highest number. The longest ROH segment of Chinese indigenous cattle was observed in Altay white-headed cattle (Supplementary Table S4). Altay white-headed cattle have lesser inbreeding than other European commercial cattle breeds but more than native Chinese cattle breeds (Figure 1B).

Regarding LD patterns, Mongolian cattle had a lower LD level at short marker distances, Altay white-headed cattle had an intermediate level and Wannan cattle had the highest level (Figure 1C). The analysis of nucleotide diversity produced results comparable to the investigation of LD (Figure 1D). Although the genetic diversity of Altay white-headed cattle is between the two clusters of indicine and taurine, it still exhibits higher levels of nucleotide polymorphisms than the taurine group.

### 3.3 Population genetic structure and phylogenetic analysis

**FIGURE 2:**
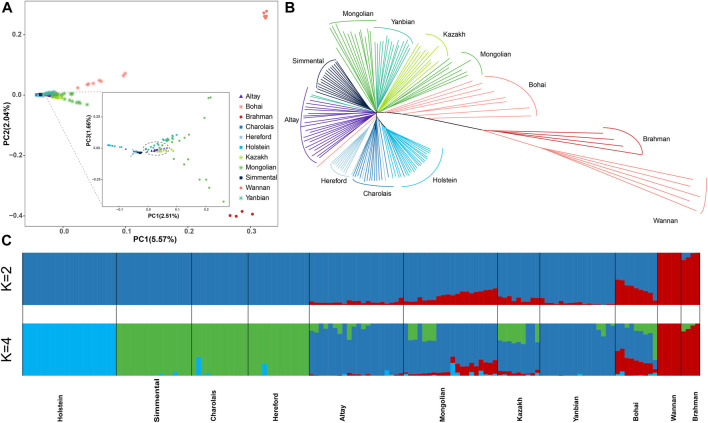
Population structure and relationships of Altay white-headed Cattle. **(A)** The plot of principal component analysis (PCA) results showing PC1 *versus* PC2. **(B)** Neighbor-joining phylogenetic tree constructed using whole-genome SNP data. **(C)** Model-based clustering of cattle breeds using ADMIXTURE with *K* = 4. Colours reflect the different cattle ancestry.

We chose representative cattle breeds from around the world based on autosomal SNPs to investigate the relationship between Altay white-headed cattle and other cattle breeds. Principal component analysis (PCA) showed distinct breed patterns, as data were kept together and grouped. PC1 distinguished taurine from indicine and PC2 distinguished taurine from Chinese and Indian indicine, explaining 5.57% and 2.04% of the overall variance, respectively (Figure 2A). In the taurine lineage, our Altay white-head cattle were closely clustered with Yanbian and Mongolian cattle and separated from European commercial cattle breeds (Simmental, Holstein, Hereford and Charolais cattle) (Figure 2A).

However, Altay white-headed cattle are found in the cluster of European cattle varieties in the phylogenetic tree (Figure 2B). We utilized admixture software to infer the individual ancestry coefficient to further identify population structure. When *K* = 2, the ancestral component predictions of all individuals separated taurine and indicine cattle lineages, when *K* = 4, taurine could be further divided into Holstein, Simmental-Hereford-Charolais and Yanbian lineages. In addition, our Altay white-headed cattle harbour the mixed ancestries of the Holstein and Simmental-Hereford-Charolais lineages (Figure 2C).

### 3.4 Candidate regions and genes under positive selection

**FIGURE 3:**
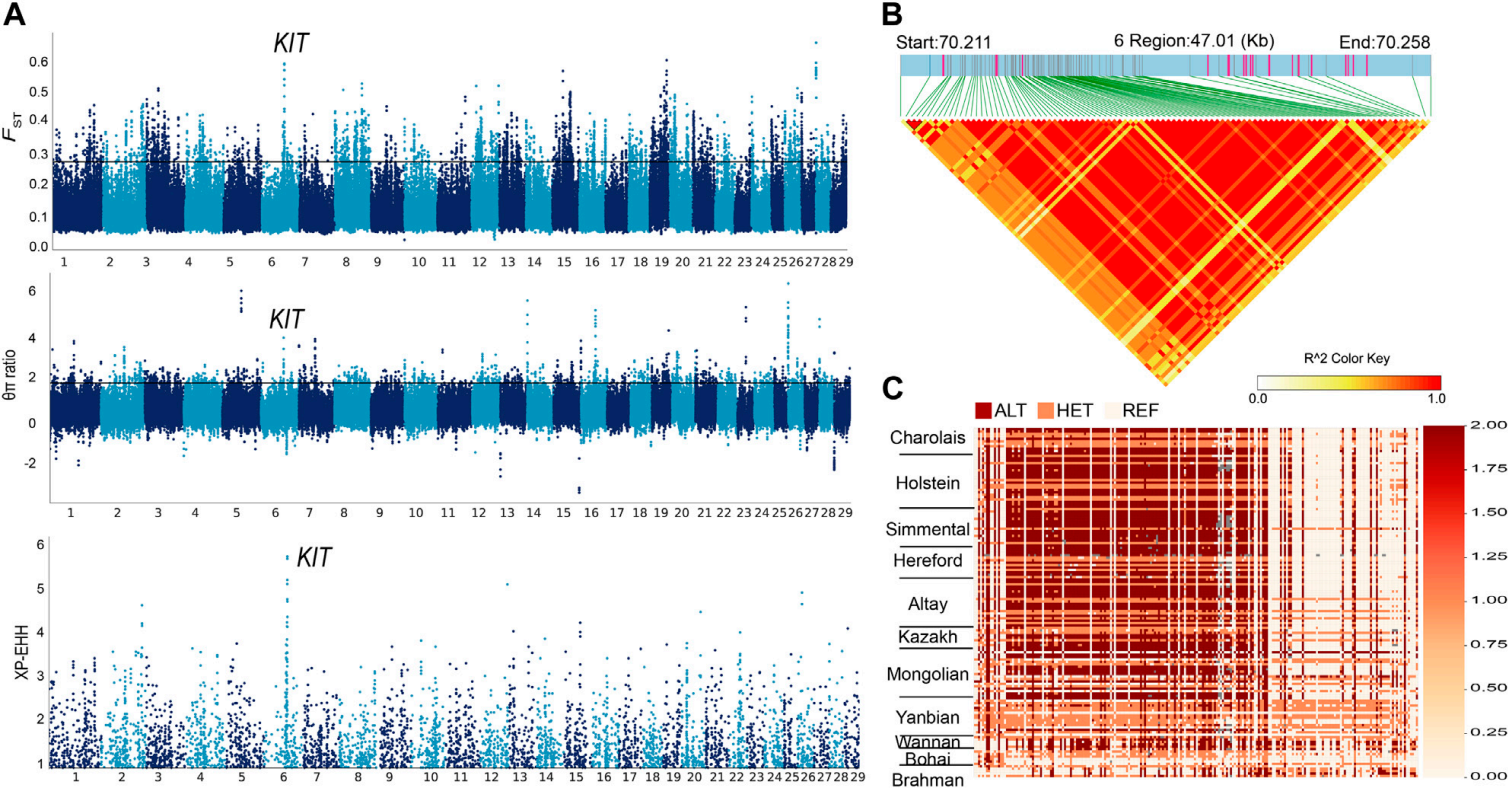
Selection signals in genomic areas of Altay white-headed cattle. **(A)** Manhattan plot of selected sweeps for Altay white-headed cattle and Bohai cattle (*F*_ST_, θπ ratio and XP-EHH) **(B)** Haplotype block in LD with Altay white-headed cattle based on 103 SNPs. **(C)** Haplotype patterns were created using SNPs with MAFs greater than 0.05. (chr6: 70.21–70.25 Mb).

Altay white-headed cattle are characterized by their white heads. We compared the genomes of Altay white-headed to Bohai black cattle (the entire coat is black) using *F*_ST_, θπ ratio and XP-EHH (Supplementary Tables S5-S7) to identify necessary selective sweeps. After merging consecutive outlier windows, 38 overlapping candidate regions were screened (Supplementary Table S8). The highest signals were located in the *KIT* region on chromosome 6 (chr6) (*F*_ST_ = 0.4, θπ ratio = 2.93 and XP-EHH = 4.81) (Figure 3A). A critical coat colour-related gene, protooncogene receptor tyrosine kinase (*KIT*), is located in this area. Linkage disequilibrium analysis in this candidate area revealed a 0.4 Mb haplotype block with full LD (LD, r^2^ = 1) (Figure 3B). The expansion of the three previously stated selection procedures to signal to the maximum point resulted in the discovery of a 47 kb region (chr6: 70.21–70.25 Mb) containing 103 SNPs (Figure 3C; Supplementary Table S9). All Altay white-headed cattle were nearly fixed in a single haplotype spanning 103 SNPs from 70,210,528 to 70,257,540 on chr6. We found that the haplotypes of Hereford cattle and those of Altay were highly similar. In addition, we focused on other potential genes that were enriched in GO terms and scanned by three selection methods between Altay white-headed cattle and Bohai black cattle. Among them, two genes (*EPB41L5*, *SCG5*) that were associated with environmental adaptability (Mbikay et al., 2001; Al-Shuhaib et al., 2018) had a strong selection sweep signal. The GO enrichment analysis uncovered multiple pathways involving critical biological processes, including " cytoplasm, GO:0005737″, and “intracellular protein transport, GO:0006886” (Supplementary Table S10).

### 3.5 Relationship inference and migration of Altay white-headed cattle

**FIGURE 4:**
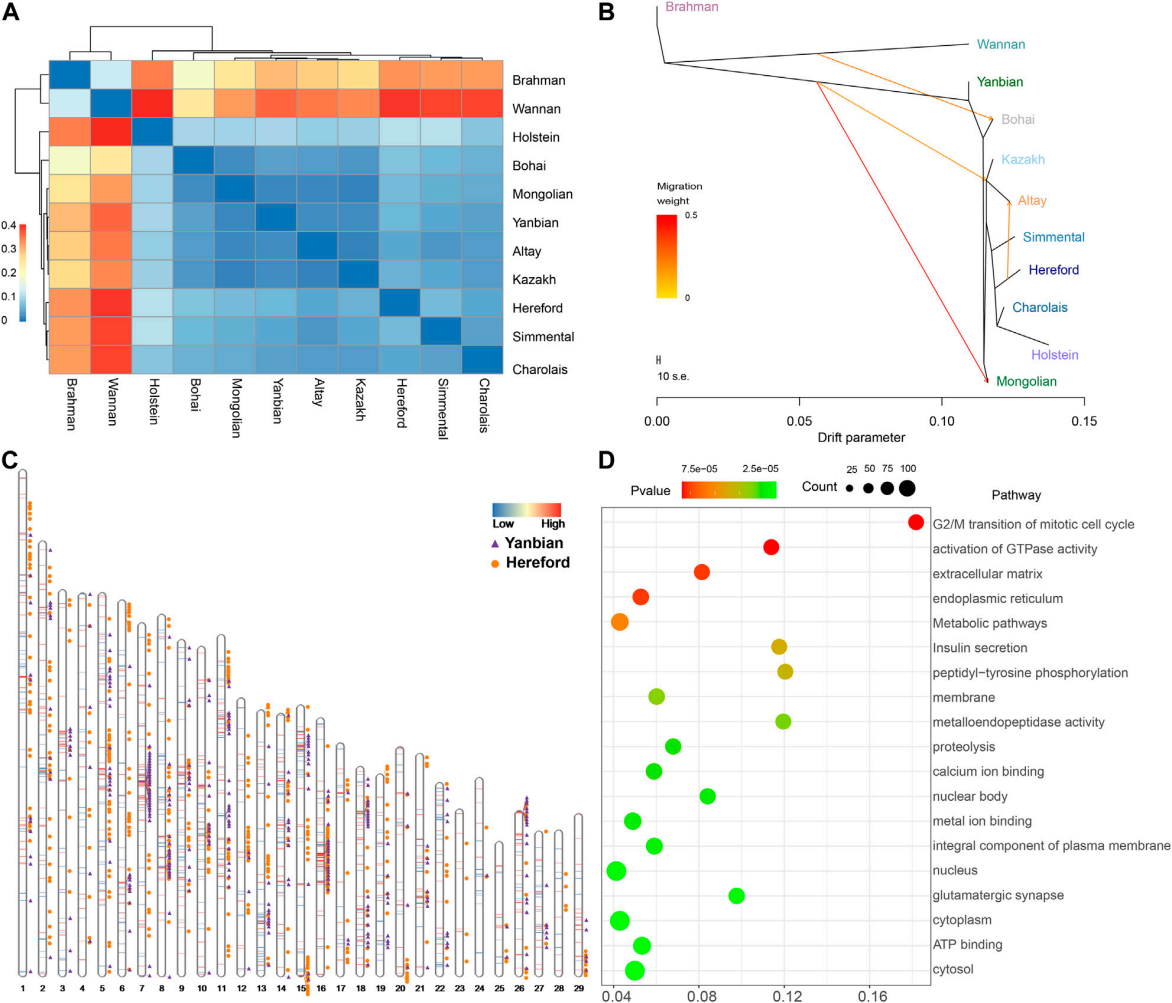
Relationship of Altay white-headed cattle and other cattle breeds. **(A)** Index of population divergence among 11 cattle breeds. **(B)** A maximum-likelihood tree is produced using TreeMix (up to migration 4). **(C)** Identification of segments in which ancestry proportions were considerably more remarkable than the percentage in the entire genome in Altay white-headed cattle. **(D)** KEGG and GO pathways derived from enrichment analysis of genes with high Hereford proportions.

The preceding admixture analysis suggested the introgression of European cattle breeds into Altay white-headed cattle (Figure 2C). We used whole-genome resequencing data from 11 cattle breeds to discover the differential genomic footprints computed by *F*_ST_ to understand better the historical relationship within the 11 populations (Figure 4A **;** Supplementary Table S11). As predicted the distinction between taurine and indicine populations is most visible and the Altay white-head cattle belong to a typical taurine cattle breed. Immediately afterwards, we determined the value of *F*_ST_ among taurine. First, the *F*_ST_ values of the Altay white-head cattle and other Chinese native cattle breeds were less than 0.05 (excluding the *Bos indicus*), second, in comparison to the European cattle breeds, the *F*_ST_ values of cattle were also less than 0.05 [Simmental (0.036), Charolais (0.037) and Hereford (0.047)], indicating that the Altay white-head cattle are closer to the native Chinese breeds than to European breeds. Migration events of Altay white-headed cattle populations were revealed by TreeMix analysis (Figure 4B **;** Supplementary Figure S1), resulting in a migration of Hereford to Altay white-headed cattle (Supplementary Table S12), consistent with the admixture results (Figure 1C), indicating a possible broad interchange of introduced European cattle breeds into Chinese indigenous cattle. We generated the *f*3 statistics for a better understanding of the possible ancestral mixtures in Altay white-headed cattle. The *f*3 statistics calculated on population triples using Altay white-headed cattle as a target, indicine cattle breeds (Brahman) and seven taurus cattle breeds as source populations resulted in a significant Z score (Supplementary Table S13). The *f*3 statistics confirmed that the Altay white-headed cattle shared the most derived polymorphisms with Hereford cattle.

Assuming this, we utilized RFMix to deduce the local ancestries of the genome of Altay white-head cattle. Most of the Altay white-head cattle segments were predicted to be Hereford and Yannbian cattle, mostly located on chromosomes 5, 7, 8, 16, and 18 (Figure 4C). Finally, we obtained 1,482 Yanbian and 2,179 Hereford segments with a high frequency (>0.9). In the 2,179 Hereford segments of Altay white-head cattle, 914 genes were identified (Supplementary Table S14). We discovered the *KIT* gene in a segment of Altay white-head cattle penetration by Hereford cattle. Many of the significantly enriched pathways (*p* < 0.05) were linked to metabolism and biological processes, such as “metabolic pathways” (bta01100, *p* = 2.844 × 10^-4^) and “peptidyl-tyrosine phosphorylation” (GO:0018108, *p* = 1.63 × 10^-4^) (Figure 4D). Furthermore, we identified 686 genes in the Yanbian cattle segment of Altay White-head cattle (Supplementary Table S15), and most pathways were implicated in “cancer pathways” (bta05200, *p* = 8.65 × 10^−5^) and “nervous system development” (GO:0007399, *p* = 7.98 × 10^-4^).

## 4 Discussion

Genetic variation lurked multifarious historical events, reflecting the historical selection and evolutionary pressures experienced as various cattle breeds developed (Ahmad et al., 2020; Kumar et al., 2021a; Kumar et al., 2021b; Ghildiyal et al., 2023). ROH is a critical metric for evaluating inbreeding and recessive inheritance (Curik et al., 2014; Nandolo et al., 2018). Altay white-head cattle have a higher ROH number than other native Chinese cattle breeds, and the length of ROH for Altay white-head cattle is greater than that of other native Chinese cattle breeds, suggesting a more noticeable inbreeding level of Altay white-head cattle in native Chinese cattle breeds. As a result, artificial insemination and other procedures are required to guarantee the stability of the population structure (Baes et al., 2019). While lower than indicine cattle and their hybrids, the nucleotide diversity of Altay white-head cattle is generally consistent with native Chinese cattle breeds. This result is consistent with previous studies and shows that indicine cattle exhibit higher levels of polymorphism than taurus cattle (Ahmad et al., 2020).

The white-headed trait of Altay white-head cattle has intrigued Xinjiang pastoralists for decades, although the genetic origin of the white-headed phenotype is still unknown. We performed a comparative population genomics analysis on the genomes of 20 Altay white-headed cattle and eight Bohai black cattle (the entire coat is black). We discovered the most important combined signals in a 47 kilobase (kb) region on chromosome 6 (chr6: 70.21–70.25 Mb) that contains a critical coat colour-related gene (*KIT*). One of the primary reasons for white coat colouration, which has been linked to *KIT*, is the reduction in melanocyte survival and dissemination. *KIT* is a tyrosine kinase that promotes melanocyte migration from the neural crest down the dorsolateral route to a final destination in the skin during embryonic development (Grichnik et al., 1998). White pattern traits are reported in various animals and have different generation methods (Xu et al., 2013; Jones et al., 2018; Cao et al., 2019; Liang et al., 2021). Given the migration of Hereford cattle and Altay white-head cattle (Figure 4C) and the *KIT* gene in a segment of Altay white-head cattle introgression by Hereford cattle (Supplementary Table S14), we speculated that the *KIT* gene might play a role in the development of the white-head phenotype in Altay white-head cattle. However, this is only a conjecture, additional theoretical and experimental confirmation is needed.

The Altay area is located in the interior of China and serves as an essential basis for cattle production. However, the rugged terrain and scant, poor-quality grazing in the winter impede the development of the local cattle business. The average winter temperature falls −10°C, with even lower temperatures ranging from−35°C to −50°C during the coldest months (Zhou et al., 2020). Functional enrichment analysis of putatively selected genes (PSGs) identified by genomic differences between Altay white-head cattle and Bohai black cattle reared in distinct contexts showed some GO terms largely linked with environmental adaptation, such as “intracellular protein transport, GO:0006886"(*p* = 7.81E-06) (Supplementary Table S10), in which the *SCG5* gene was responsible for ostrich adaptation (Al-Shuhaib et al., 2018). Secretogranin V (also known as neuroendocrine protein 7B2), which has 208 amino acids in ostriches, is encoded by the *SCG5* gene. This acidic protein, found in the secretory granules of neuroendocrine cells, participates in the secretory route *via* which zymogen is activated and developed and is functionally related to ostrich adaptation (Mbikay et al., 2001; Al-Shuhaib et al., 2018). We also discovered that *EPB41L5* (GO:0005737, *p* = 1.65E-19) (Supplementary Table S10) were linked to the integrity of renal filtration (Guo et al., 2020; Maier et al., 2021). *EPB41L5* (Erythrocyte Membrane Protein Band 4.1 Like 5) is a protein-coding gene. The extracellular matrix receptor *EPB41L5* ensures the mechanical stability required for podocytes at the kidney filtration barrier by linking to extracellular matrix sensing and signalling, focal adhesion maturation, and actomyosin activation (Schell et al., 2017; Maier et al., 2021). Therefore, it is reasonable to think that *EPB41L5* is important for the survival of cattle in dry environments.

Altay white-headed cattle are closely connected to other Chinese indigenous taurus cattle breeds (Inner Mongolia, Kazakh, and Yanbian), as evidenced by PCA and ancestry estimation (Figures 2A, C), In contrast, we also discovered that it contained the ancestral lineage of European cattle breeds (Figure 2B). This intriguing discovery motivated us to investigate the ancestry admixture of Altay white-headed cattle carefully. The outcomes of relationship inference and migration analysis were the same: there was genetic infiltration of Hereford cattle into Altay white-head cattle (Figure 4B; Supplementary Table S13). We used RFMix to infer local ancestry in our investigation. A series of large fragments of annotated genes involved in critical biological processes (Supplementary Tables S14-S15) may reflect adaptation to the local environment and artificial selection during the development of Altay white-head cattle, making genomic information more accessible for local ancestral inference about admixture processes.

## 5 Conclusion

Compared to other Chinese indigenous cattle, higher inbreeding suggested an endangered status. While principal component and admixture analysis showed closer to other Chinese taurine, migratory events of Hereford cattle into Altay white-headed cattle were also detected, leading us to speculate that the white-headed phenotype of Altay white-headed cattle is connected to Hereford cattle. Furthermore, we discovered several candidate genes responsible for Altay white-headed cattle environmental adaptation. These findings lay the groundwork for future studies into the genomic properties of other major indigenous cattle breeds.

## Data Availability

The original contributions presented in the study are publicly available. This data can be found in the NCBI (https://dataview.ncbi.nlm.nih.gov/object/PRJNA917513?reviewer=mcaku5nh3n45c8gi2209f0c82g). Accession number: PRJNA91751.
