# Supplementary figures and images for: Genome-wide analysis emancipates genomic diversity and signature of selection in Altay white-headed cattle of Xinjiang, China
Source: Front Genet. 2023 Mar 30;14:1144249. doi: 10.3389/fgene.2023.1144249 (PMC10098193; doi:10.3389/fgene.2023.1144249)

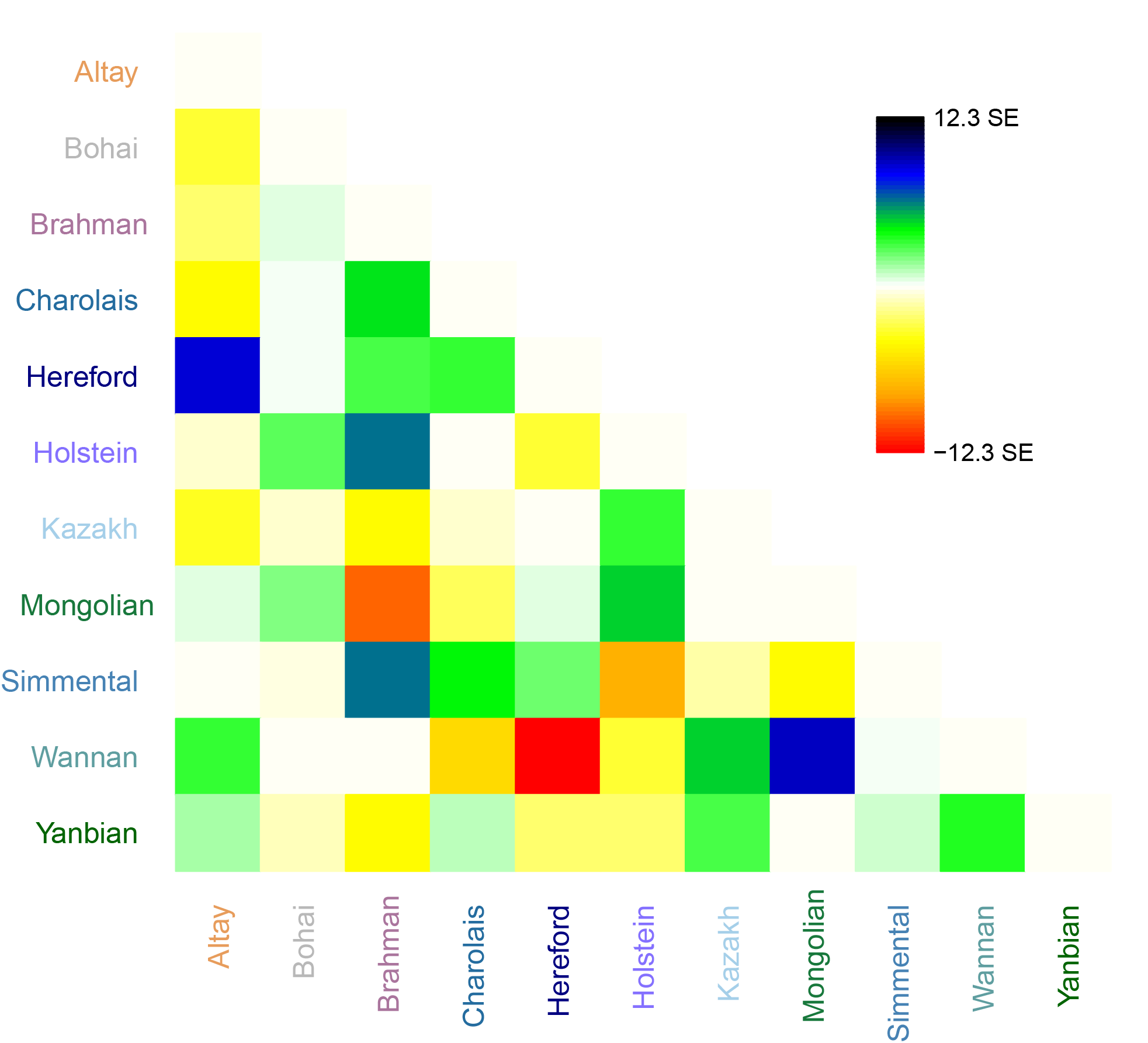

Supplement: Supplementary file 1 [file Image1.TIF]
